# Towards the smart and sustainable transformation of *Reverse Logistics 4.0*: a conceptualization and research agenda

**DOI:** 10.1007/s11356-022-22473-3

**Published:** 2022-08-16

**Authors:** Xu Sun, Hao Yu, Wei Deng Solvang

**Affiliations:** Department of Industrial Engineering, UiT—The Arctic University of Norway, Lodve Langesgate 2, 8514 Narvik, Norway

**Keywords:** Industry 4.0, Technological transformation, Smart technologies, Reverse supply chain, Waste management, Sustainability

## Abstract

The recent advancement of digitalization and information and communication technology (ICT) has not only shifted the manufacturing paradigm towards the Fourth Industrial Revolution, namely Industry 4.0, but also provided opportunities for a smart logistics transformation. Despite studies have focused on improving the smartness, connectivity, and autonomy of isolated logistics operations with a primary focus on the forward channels, there is still a lack of a systematic conceptualization to guide the coming paradigm shift of reverse logistics, for instance, how “individualization” and “service innovation” should be interpreted in a smart reverse logistics context? To fill this gap, *Reverse logistics 4.0* is defined, from a holistic perspective, in this paper to offer a systematic analysis of the technological impact of Industry 4.0 on reverse logistics. Based on the reported research and case studies from the literature, the conceptual framework of smart reverse logistics transformation is proposed to link Industry 4.0 enablers, smart service and operation transformation, and targeted sustainability goals. A smart reverse logistics architecture is also given to allow a high level of system integration enabled by intelligent devices and smart portals, autonomous robots, and advanced analytical tools, where the value of technological innovations can be exploited to solve various reverse logistics problems. Thus, the contribution of this research lies, through conceptual development, in presenting a clear roadmap and research agenda for the reverse logistics transformation in Industry 4.0.

## Introduction

Recently, the increasing focus on sustainable development and circular economy from the whole society and the more stringent environmental regulations have required companies to take responsibility for the entire lifecycle of their products. The primary aim of reverse logistics is to maximize the recovery of the remaining value from end-of-life (EOL) products through the proper design, operating, controlling, and maintaining effective and economic-efficient flows starting from customers towards initial suppliers and manufacturers (Rogers and Tibben-Lembke 2001), and the non-recyclables should be appropriately disposed of. Designing and operating a reverse logistics system need to balance the trade-off between economic, environmental, and social sustainability (Ramos et al. 2014). However, this is not an easy endeavor due to the complexity of effectively managing several stakeholders to perform various operations including collection, sorting, distribution, disassembling, repair, reuse, remanufacturing, recycling, energy recovery, and proper waste disposal (de Paula et al. 2019). Furthermore, the increased system operating costs (Plaza-Úbeda et al. 2021), the high uncertainty related to the quantity and the quality of EOL products in the reverse flows (Trochu et al. 2018), the lack of relevant and real-time information for decision-making (Liu et al. 2019;Wang and Wang 2019), and the lack of coordination among different partners (Plaza-Úbeda et al. 2021) have become some major obstacles for sustainable reverse logistics management.

These challenges may be better tackled today with the emerging concept of Industry 4.0 as well as its enabling technologies, which provide new opportunities for achieving improved internet-based connectivity, smartness, intelligence, and autonomous operations of not only manufacturing processes but also logistics systems (Bai et al. 2020;Sarkis et al. 2020). Taking advantage of the technological innovation of the Fourth Industrial Revolution, the concept of Logistics 4.0 has also been proposed in recent years (Wang 2016;Winkelhaus and Grosse 2020). Combining several cutting-edge technologies, e.g., internet of things (IoT), big data analytics, and artificial intelligence (AI), in a cyber-physical system (CPS) that integrates both computational intelligence and smart physical assets, a Logistics 4.0 system can achieve real-time monitoring and decision-making, responsive communications, better resource allocation, and smoother material flows. These smart technologies can also be used to improve the economic, environmental, and social sustainability of reverse logistics systems.

The changing demands and the integration of different Industry 4.0 technologies will together lead to a paradigm shift of reverse logistics, where the former is the driver and the latter is the enabler of this smart and sustainable transformation. The increased data availability can improve the prediction and traceability of EOL products, which minimizes the uncertainty of the reverse flows and improves the planning of different operations, e.g., collection (Sung et al. 2020) and remanufacturing (Kerin and Pham 2019;Wang and Wang 2019). The high-quality data also improves the outputs of the model-based optimization and simulation approaches for critical decisions (Liu et al. 2019), i.e., scheduling of collection, routing, inventory management, and distribution. In addition, the increased use of AI-enabled smart robots can replace human workers in the harsh working environment, and the enhanced interaction between different partners and stakeholders via a highly connected digital platform may improve inter-company information sharing and resource utilization.

Even though recent studies have been conducted to show the application of several Industry 4.0 technologies in isolated reverse logistics operations, there is still a lack of a systematic conceptual framework to better understand the potential and implications of these technological innovations for the entire reverse logistics system, particularly from the service innovation perspective. For example, how “individualization” should be interpreted in a smart reverse logistics context? Furthermore, most studies only emphasize the benefits of implementing Industry 4.0, but much less effort has been paid to discussing the challenges of technological adoption in reverse logistics systems. Therefore, by analyzing the state-of-the-art research and case studies in a comprehensive and cross-disciplinary manner, this paper aims at filling these gaps by answering the following three research questions (RQs):

- *RQ1*: What are the definition and the key features of *Reverse Logistics 4.0*?
- *RQ2*: What is the smart and sustainable transformation of *Reverse Logistics 4.0*?
- *RQ3*: What is the future research agenda of *Reverse Logistics 4.0*?

By answering these questions, we define the concept of *Reverse Logistics 4.0* in comparison with the four Industrial Revolutions in history, where the technology-enabled innovation in both service and operations is systematically analyzed in the reverse logistics context. Moreover, based on the reported research and case studies from the literature, we present a roadmap for both researchers and practitioners in the smart and sustainable reverse logistics transformation. Finally, we also present a research agenda in four directions: (1) smart and innovative reverse logistics services; (2) quantitative models for smart and sustainable reverse logistics management; (3) digital reverse logistics twin, and (4) human-centricity and *Reverse Logistics 5.0*.

The rest of the paper is organized as follows. The “State of the art” section provides state-of-the-art developments in both reverse logistics and Industry 4.0. The “Reverse Logistics 4.0” section conceptualizes *Reverse Logistics 4.0* and discusses its main features. The “Smart and sustainable reverse logistics transformation” section investigates the smart and sustainable reverse logistics transformation enabled by disruptive technologies. The “A future research agenda” section identifies a future research agenda. The “Conclusion” section concludes the paper.

## State of the art

### Reverse logistics

Reverse logistics focuses on the value recovery from EOL products and on the proper treatment of non-recyclables (Rogers and Tibben-Lembke 2001). The reuse and recycling practices can be dated back to a long time ago, for example, after proper cleaning and treatment, the returned bottles can be reused many times by beverage manufacturers for their new products. In the early 1990s, the concept of reverse logistics was first put forward to depict all relevant activities and logistics flows from the end customers to different producers and recyclers as well as other actors (Salema et al. 2007). The main operations of a reverse logistics system consist of the collection of EOL products from customers and end-users, the appropriate inspection, sorting, disassembling and/or pre-processing, the distribution of different products, parts and components to respective facilities for proper treatment, and the planning and scheduling of facility operations and transportation (Agrawal et al. 2015; Fleischmann et al. 1997). Configuring a reverse logistics system for effective management of these operations requires proper decision-making at strategic, tactical, and operational levels. During the past three decades, extensive research efforts have been spent to improve conceptual development (Dowlatshahi 2000; Lambert et al. 2011), formulate advanced mathematical models and algorithms (Diabat et al. 2013; Govindan et al. 2016), provide empirical studies and implications (Waqas et al. 2018), and develop other qualitative and quantitative methods for supporting various decisions in reverse logistics (Govindan and Bouzon 2018).

The motivation of reverse logistics comes initially from two aspects (Fleischmann et al. 1997). From the ecological perspective, reverse logistics can improve the utilization of different materials and can thus help to solve the global resource depletion problems. Besides, it may provide companies with opportunities to improve their cost reduction and profitability through product recovery. However, in practice, the value recovery through reverse logistics may be drastically hindered by several factors, i.e., the low-profit margin (Ravi and Shankar 2015), the possible competition with new products or market cannibalization (Atasu et al. 2010), the uncertainty related to market acceptance (Calvo-Porral and Lévy-Mangin 2020), and the complexity of managing reverse flows. Moreover, even though reverse logistics has been considered a fundamental part of sustainable development and circular economy, improper recycling activities may result in negative environmental and social impacts (Julianelli et al. 2020). For example, the large export volume of waste electrical and electronic equipment (WEEE) from developed countries, i.e., the USA, EU, and Japan, to the developing countries in southeast Asia not only causes increased greenhouse gas (GHG) emissions related to maritime transportation but also poses significant threats to the workers and the environment due to the primitive and low-tech recycling methods used. Thus, the effective design of a reverse logistics system will help to promote more sustainable practices of different activities.

**Fig. 1:**
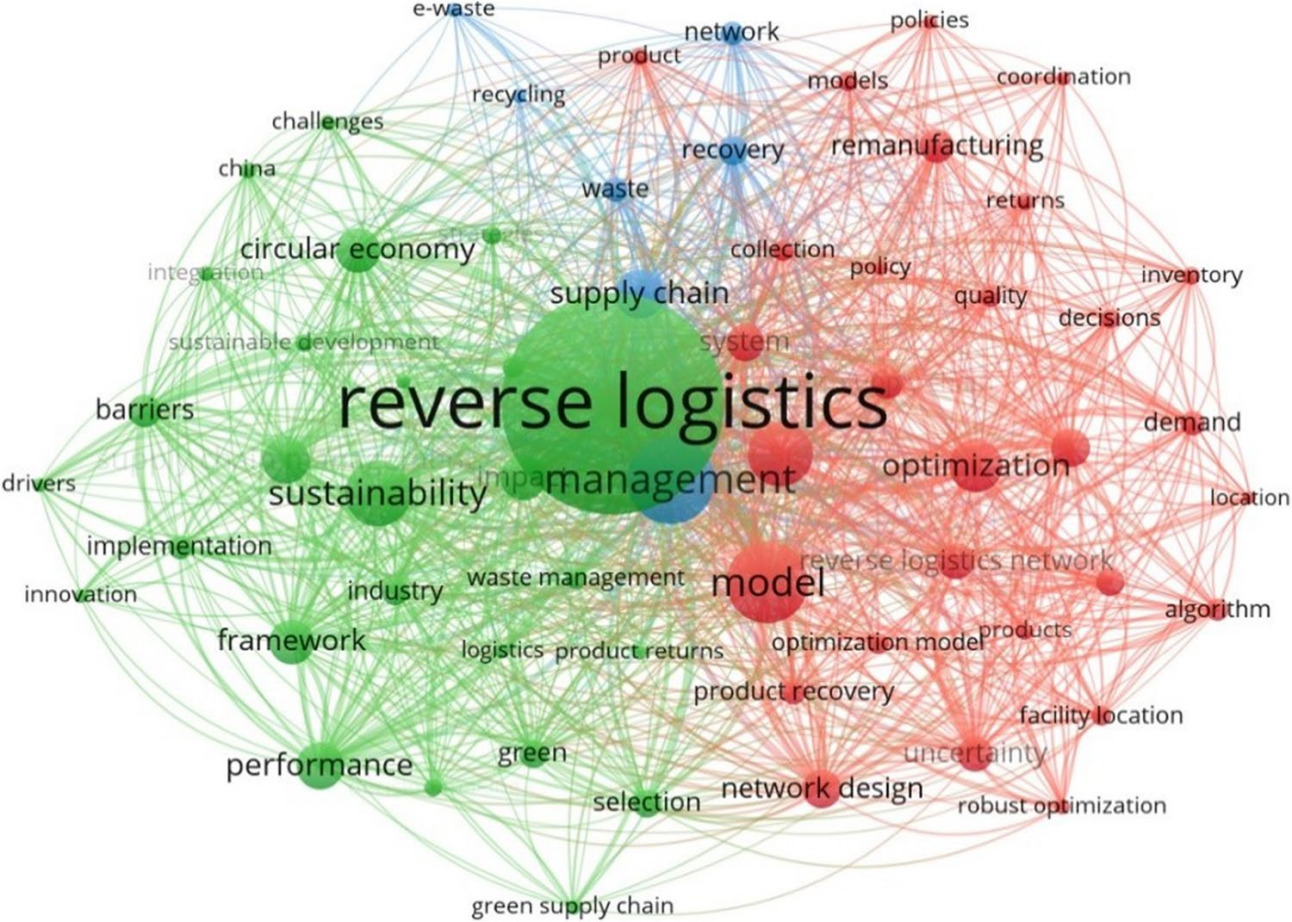
Keyword co-occurrence analysis of reverse logistics

Figure 1 presents a keyword co-occurrence analysis of the latest publications on reverse logistics. The web of science (WOS) database was used for searching the relevant papers to generate the visualization. The recent research on reverse logistics has focused on managing various types of EOL products through different options considering economic, social, and environmental performances. Several important decisions, i.e., facility location, transportation, and vehicle routing, have been predominantly tackled by using advanced quantitative methods, i.e., mathematical models (Govindan et al. 2015), multi-criteria decision support methods (Senthil et al. 2018), and simulation (Beiler et al. 2020;Gonçalves et al. 2019;Pandian and Abdul-Kader 2017). Among these, optimization is the most extensively used technique to solve complex decision-making problems in reverse logistics. Early research focuses on developing deterministic single objective optimization models for either minimizing the system operating cost or maximizing the total profit (Govindan et al. 2015). However, recent studies emphasize the balance among different sustainable indicators with multi-objective optimization models (Govindan et al. 2016;Yu and Solvang 2018), the proper formulation and treatment of uncertainties (Fattahi and Govindan 2017;Soleimani et al. 2021;Yu and Solvang 2016), the improvement of the models’ computational efficiency (Afra and Behnamian 2021;Alshamsi and Diabat 2017), and the management of different stakeholders (Gu et al. 2019).

### Industry 4.0

Industry 4.0, also known as the Fourth Industrial Revolution, was put forward by German researchers and industrial practitioners in 2011 (Frank et al. 2019), which presented the blueprint of the next-generation manufacturing systems with the adoption of state-of-the-art manufacturing technologies and ICT. Even if it is a new concept, Industry 4.0 has been widely discussed by worldwide researchers during the last decade due to its potential to dramatically change today’s paradigms of almost all industries and businesses through digital transformation. On the one hand, the current change in the industrial paradigm is driven by new demands for increased individualization on both products and services, shortened time-to-market, small-scale decentralized customer segments, and so forth. On the other hand, these new demand patterns can be better addressed with recent technological advancements that have provided companies with opportunities to achieve a highly flexible, agile, responsive, and resource-efficient manufacturing process through digitalization and various smart technologies (Lasi et al. 2014). Compared with the Third Industry Revolution starting from the early 1970s, where industrial robots, advanced machine tools, computer-aided manufacturing (CAM), and lean production were used to achieve mass customization through increased automation, reconfigurability, and flexibility, Industry 4.0 has several new features. From the technological perspective, an Industry 4.0 manufacturing system emphasizes the internet/5G-based communication and connectivity of different smart devices and cyber elements, which enable real-time data collection, autonomous system control, and effective human-machine interaction (Salkin et al. 2018). Another significant feature is the computational intelligence brought by AI, big data analytics, and improved optimization and simulation tools, which enables better prediction and real-time data-driven decision-making. From the commercial perspective, these Industry 4.0 technologies pave the way for new business models, individualized customization, better resource sharing, and sustainable production (Bag et al. 2021;Bai et al. 2020).

Based on Salkin et al. (2018), Bai et al. (2020), Frank et al. (2019), and Phuyal et al. (2020), Figure 2 summarizes the key Industry 4.0 technologies into three categories, namely, physical layer, cyber layer, and cyber-physical layer. The drastically increased use of connected devices has driven a rapid digital transformation. Recent research has shown that the total amount of connected devices in the world has increased by nearly 99 times during the past two decades, and the average number of connected devices per person has reached approximately 6.58 in 2020 (Phuyal et al. 2020). An Industry 4.0-enabled manufacturing system comprises a large amount of various smart and connected robots and devices, which are communicated with each other and interacted with cyber intelligence in real-time. The level of integration of both physical elements and cyber technologies within a CPS determines the system’s sophistication, connectivity, intelligence, and autonomy. Lee et al. (2015) defined five levels of technological integration in a CPS, which are machine-level connection, data transmission and conversion, system-level connectivity, system cognition, and system intelligence and self-configuration. With the highest level of CPS, a smart manufacturing system can make self-decisions based upon individual customer orders, generate production procedures, test different scenarios in virtual environments, and control intelligent robots and machines for an autonomous and highly responsive production process.

**Fig. 2:**
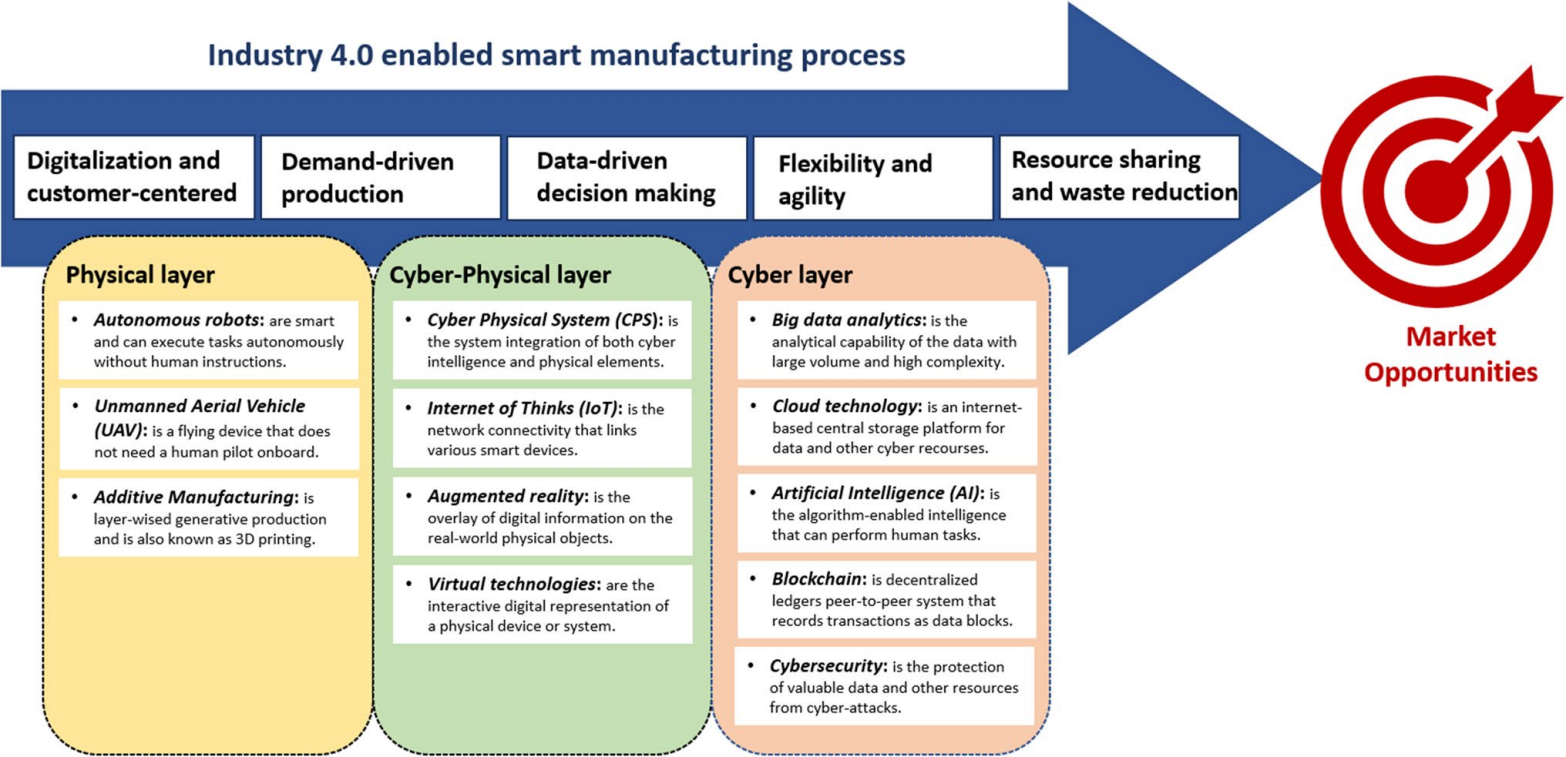
Industry 4.0-enabled smart manufacturing

**Fig. 3:**
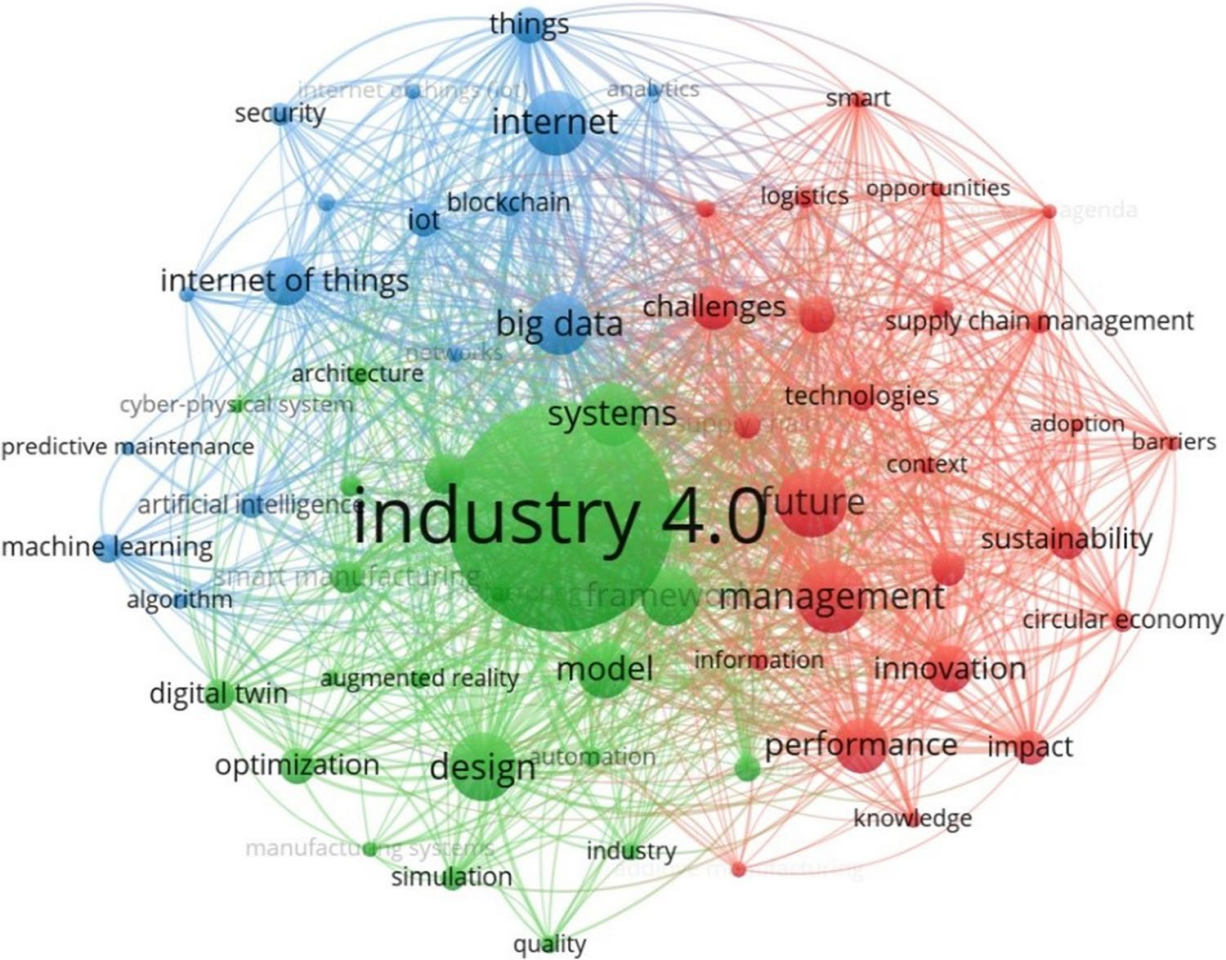
Keyword co-occurrence analysis of Industry 4.0

Figure 3 illustrates the keyword co-occurrence analysis of the recent research related to Industry 4.0. The research focuses have been predominantly given to the technological development of CPS, IoT, AI, big data analytics, blockchain, additive manufacturing, etc., to achieve predictive maintenance, real-time decision-making, smart manufacturing, and better production control and planning. Besides, these technologies are not only used to improve manufacturing processes but are also applied to enhance supply chain management (Fallahpour et al. 2021), innovation (Liu and De Giovanni 2019), and sustainable development (Bradu et al. 2022). Particularly, recent research has shown great opportunities to improve sustainability and circular economy with the help of Industry 4.0 (Bag et al. 2018;Bai et al. 2020), for example, reducing waste generation and improving material utilization by adopting a demand-driven small-scale intelligent production process with additive manufacturing (Ford and Despeisse 2016).

### The contributions of this research

Table 1 shows the search information of the top 60 keywords related to reverse logistics and Industry 4.0, and the top keywords related to both reverse logistics and Industry 4.0 are “sustainability,” “circular economy,” “model,” “impact,” “challenges,” “systems,” “design,” and “sustainable development,” respectively. Through a comparison of these keywords, it is noteworthy that even if Industry 4.0 has provided new opportunities for improving decision-making and operations with better use of smart devices, data analytics, and computational intelligence, their adoption in reverse logistics is still in its infancy and has not been widely discussed in the literature. For example, many optimization models have been developed for supporting decision-making in reverse logistics, whose results are heavily dependent on the availability and the quality of input data. The high uncertainty related to the input parameters will make these models computationally expensive to solve within polynomial time. Moreover, the reliability of the models’ outputs and the decisions obtained may also be drastically influenced. Due to these, the adoption of Industry 4.0 technologies is well justified for their impacts on improving the data quality, computational intelligence, and operations in reverse logistics.

**Table 1:** Literation analysis of the recent publications

Search criteria	Keywords		
	“Reverse logistics”	“Industry 4.0”	“Reverse logistics” AND “Industry 4.0”
Database	Web of Science	Web of Science	Web of Science
Source	Journal	Journal	Journal
Language	English	English	English
Total articles	1282	5135	21
Total keywords	4704	16575	174
Co-occurrence threshold	35	70	3
Selected keywords	62	63	16

Based on both theoretical and practical insights related to different reverse logistics activities, this paper aims at providing a systematic conceptual development and research agenda for the smart and sustainable transformation of reverse logistics in Industry 4.0, namely *Reverse Logistics 4.0*. The contributions of this paper can be summarized as follows:

- The concept of *Reverse Logistics 4.0* is defined considering both technological advancement and service innovation.
- The conceptual framework for smart and sustainable *Reverse Logistics 4.0* transformation is formulated.
- The research agenda for smart and sustainable *Reverse Logistics 4.0* transformation is given.

## Reverse Logistics 4.0

This section first introduces the conceptual development of Logistics 4.0, based on which the concept of *Reverse Logistics 4.0* is defined.

### Logistics 4.0

Today, the phrase “4.0” has been widely used not only in the manufacturing industry but also in many other fields to describe the future paradigm shifts brought by digitalization and advanced ICT. By adopting the technological innovations from Industry 4.0, the concept of Logistics 4.0 was first put forward in 2014 (Akinlar 2014), which emphasized the real-time ability, fast decision supports, and convertibility of a new IT system empowered by CPS for supporting logistics decisions. Concerning the four Industrial Revolutions in history, Wang (2016) systematically summarized the four logistics evolution stages featured with the mechanization of transportation (Logistics 1.0), the automation of logistics operations (Logistics 2.0), the advancement of logistics management systems (Logistics 3.0), and the smart and autonomous logistics systems (Logistics 4.0), respectively. Several researchers argue that Logistics 4.0 is to digitize and automize the logistics processes and operations with the help of CPS (Barreto et al. 2017), whose technological architecture requires six layers, namely, the physical asset layer, the data acquisition layer with sensors and middleware, the control layer, the database layer, the analytical and decision support layer, and the management layer (Wang 2016). From the business innovation perspective, Logistics 4.0 is viewed as a conceptual extension of Industry 4.0, whose main features are discussed in several studies (e.g., Yu and Solvang (2017))):

- *Demand-driven individualization and personalization*: Value proposition by satisfying highly individualized customer demands with CPS, customer-involved design, additive manufacturing, and pull production and logistics.
- *Product-service system*: Transforming towards the increased selling of services instead of the selling of products, for example, Rolls-Royce’s TotalCare^®^ program, also known as Powered-by-the-hours, has helped to achieve a win-win solution for both the airlines and the jet engine manufacturer (Smith-Gillespie et al. 2018).
- *Digitalization*: Increased digitalization enables effective communication between humans and machines, and it helps to converge the physical and virtual worlds.
- *Autonomous operations*: Different logistics operations, e.g., material handling and transportation, will become increasingly autonomous with the help of IoT, CPS, AI, UAV, and smart robots.
- *Resource sharing*: The real-time data collection and analytical power enabled by IoT, AI, and advanced optimization improve the level of resource sharing among different stakeholders in a logistics system, which may offset the increased cost and environmental impacts to satisfying small-scale individualized and geographically dispersed customer demands with a high service level.
- *Green and sustainable logistics*: The waste generation can be reduced with on-demand and additive manufacturing, and the environmental impacts of various logistics operations can be better tracked and minimized with blockchains.

To achieve these goals, increased digitalization and system integration at both intra- and inter-enterprise levels are required to facilitate effective interactions among stakeholders, better use of data, real-time decision-making, streamlined operations, and improved resource utilization in a logistics system. Recently, the conceptual development of Logistics 4.0 trends to synchronize business innovations with technological advancements, where business innovations are considered the goals of the next generation of smart logistic systems and technological advancements are believed to be the enablers to realize these goals. As defined by Winkelhaus and Grosse (2020), Logistics 4.0 refers to cost-affordable and highly responsive logistics services for individualization and personalization empowered by smart technologies. To further facilitate the adoption of the concept of Logistics 4.0, studies have been conducted to provide implications on the use of different Industry 4.0 technologies in various logistics operations (Sun et al. 2021), to establish models for measuring the maturity level of Logistics 4.0 (Facchini et al. 2020;Oleśków-Szłapka and Stachowiak 2018), and to understand the relevant human factors and learning effects (Wrobel-Lachowska et al. 2017).

### Reverse Logistics 4.0

Even though Logistics 4.0 has been increasingly discussed in recent years, not as much research focus has been given to reverse logistics (Sun et al. 2021). On the one hand, several Industry 4.0 technologies can benefit reverse logistics operations in the same way as they do in forward logistics. However, on the other hand, there are significant differences between forward logistics and reverse logistics in terms of their purposes and operations. For instance, the business objective of a Logistics 4.0 system is to achieve the value proposition through providing highly individualized products and responsive services, but for a reverse logistics system, the purpose may be different or the meaning of individualization may need to be interpreted in another way, for example, an individualized collection schedule in a smart waste management system. Thus, it is important to provide a thorough understanding of *Reverse Logistics 4.0*.

**Fig. 4:**
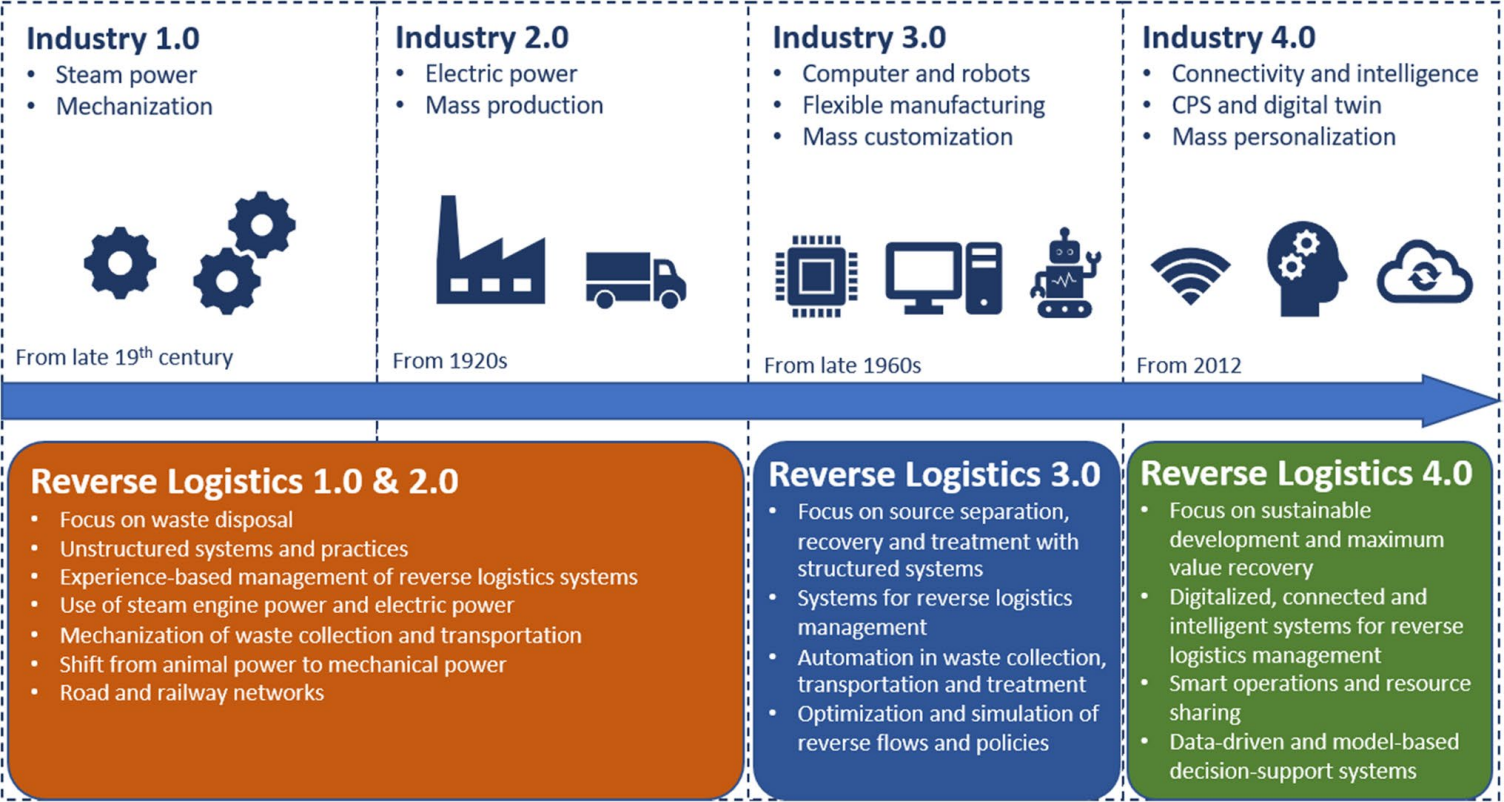
Reverse logistics evolution compared to the four Industrial Revolutions

Recently, increasing efforts have been spent to improve the sustainability and the operations of reverse logistics with Industry 4.0 technologies, for example, through real-time information sharing and diffusion of green products (Dev et al. 2020a; Dev et al. 2020b). From the conceptual development perspective, Fig. 4 presents a systematic paradigm change of reverse logistics with respect to the four industrial revolutions. Even though reverse logistics was not conceptualized before the early 1990s, its activities were widely practiced, e.g., part recycling and waste disposal. The modern industrialization from the early nineteenth century led to an increase in population and rapid urbanization, which created the market for second-hand products and raised the need for modernized reverse logistics systems. An early organized material recycling and waste management system was established in London, UK, to maintain sanitation and the general quality of urban life (Velis et al. 2009). Similar to the impacts in other industries, the first two industrial revolutions changed the means of collection, transportation, and disposal of waste with mass mechanization and the use of steam power and electricity. However, the main destinations of used products were either second-hand markets or dumpsites, and well-organized recycling activities were not widely practiced at that time. With the increased concerns on environmental pollution and resource depletion, the focus of reverse logistics shifted from waste landfill to resource recovery through better source separation and increased reuse, remanufacturing, and recycling activities. The advancements of computers and robotics in Industry 3.0 helped to better support decision-making with advanced optimization, simulation, and geographical information system (GIS) and to automize various reverse logistics operations. Besides, the drastically increased reconfigurability and flexibility of manufacturing systems not only realize mass customization but also pave the way for flexible remanufacturing in reverse logistics (Duberg et al. 2020). In this period, diverting the EOL product flows from landfills to other value recovery alternatives was the focus, and reverse logistics was conceptualized to depict all relevant activities and flows related to the effective management of EOL products (Fleischmann et al. 1997).

During the last decade, not only the economic benefits from product recovery but also the environmental and social performances of the entire reverse logistics system have been increasingly focused on through a holistic trade-off analysis (Govindan et al. 2016). Besides, the technological advancements have provided digital and smart solutions to change the paradigms of reverse logistics mainly in three ways: data, services, and operations. The value of data has been unprecedently uncovered by adopting IoT, smart devices, AI, and big data analytics, which enable better and real-time planning of different resources and operations. The cloud-based interactive and intelligent digital platform connects different service providers and customers to achieve optimal resource sharing and provide innovative services. Consumers’ involvement in reverse logistics has become increasingly important (Plaza-Úbeda et al. 2021), which provides better information on the quality, quantity, time, and location of return of different EOL products (Wang et al. 2014; Wang and Wang 2019). Furthermore, reverse logistics activities become increasingly autonomous with the use of AI-supported smart robots and vehicles. Thus, based on these characteristics, the concept of Reverse Logistics 4.0 is defined as follows:

*Reverse Logistics 4.0 is the sustainable management of all relevant flows and activities for value recovery and/or proper disposal of EOL products by using data-driven and smart technologies enabled individualization and innovative services.*

Compared with the current definitional elements, *Reverse Logistics 4.0* emphasizes the use of data and smart technologies to realize innovative reverse logistics services and to achieve harmony among the three pillars of sustainable development including economic effectiveness, environment friendliness, and social responsibility. In the context of *Reverse Logistics 4.0*, the phrase “individualization” represents service smartness and innovations, whose demands are either pulled by customers, e.g., individualized collection (Sung et al. 2020), or driven by product and data, e.g., data-driven remanufacturing of WEEE (Wang and Wang 2019). For example, an individual collection and remanufacturing process can be planned and optimized based on the real-time information of the EOL product flows, e.g., type of product, material, structure, and quality level, and the available resources of the company.

## Smart and sustainable reverse logistics transformation

**Fig. 5:**
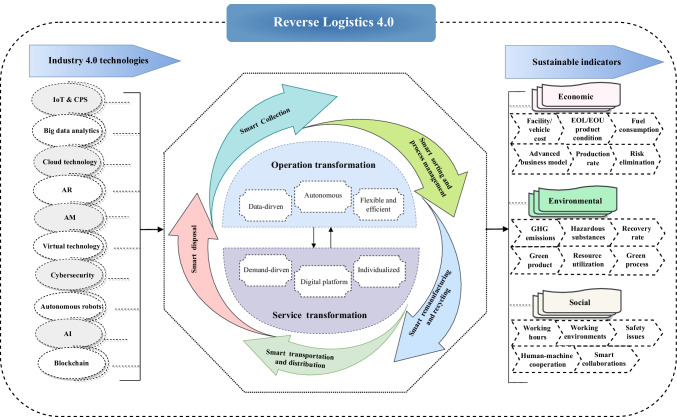
A conceptual framework of smart and sustainable reverse logistics transformation in *Reverse Logistics 4.0*

Based on the definition of *Reverse Logistics 4.0*, Fig. 5 presents a conceptual framework for smart reverse logistics transformation, where the role of Industry 4.0 technologies in shaping the reverse logistics service and operations and the three pillars of sustainable development are focused on. The conceptual framework consists of four fundamental elements that drive the paradigm transition in *Reverse Logistics 4.0*:

1. The key Industry 4.0 technologies, e.g., IoT, CPS, AI, and autonomous robots, are enablers to support the smart reverse logistics transformation.
2. The five main reverse logistics processes, i.e., collection of EOL products, sorting and pre-processing, transportation, value recovery through remanufacturing and recycling, and disposal, are affected by adopting disruptive technologies.
3. The improvement in the reverse logistics service and operations is centered on the reverse logistics transformation.
4. The targeted areas in the triple-bottom line for improving the economic, environmental, and social sustainability in reverse logistics.

This conceptual framework explicitly illustrates the connections between technological enablers, reverse logistics processes and transformations, and sustainability goals. With the increasing adoption of Industry 4.0 technologies, the transformation of reverse logistics service and operations is centered on *Reverse Logistics 4.0*:

- *Smart service transformation*: Demand-driven and service-oriented transformation is the key driver of *Reverse Logistics 4.0*. For example, individualized collection services of used products can be provided to maximize the customer value and service. However, providing such kind of service in a traditional reverse logistic system is usually expensive and requires much more resource commitments. Thus, a digitalized platform may enhance better communication and information sharing among different stakeholders in real-time, and a data-driven intelligent decision support system may help to improve resource planning and utilization, based on which individualized services can be performed in an efficient manner.
- *Smart operation transformation*: Data-driven and autonomous operations are the key enablers of *Reverse Logistics 4.0* to offset the increased costs of providing a high level of individualized service, improve the operational effectiveness and resource efficiency, minimize downtime, reduce the risks and harshnesses in the working environment, and so forth. For example, the collection, transportation, and remanufacturing of used products can be better planned with both predictive data and real-time data. Besides, the operations and working environment of various reverse logistics activities can be potentially improved, e.g., autonomous and highly accurate waste sorting with AI-enabled smart robots.

The logic of this conceptual framework indicates that, essentially, the smart service and operation transformations across all stages of a reverse logistics system are driven by the better meeting of the targeted sustainability goals, while on the other hand, Industry 4.0 technologies are the most important enablers. It is noteworthy that, in this smart paradigm transition, adopting new and disruptive technologies is not the goal but rather the means to enable responsive services and efficient processes. Meanwhile, technology itself will not lead to the better system performance of a reverse logistics system, but the transformation and redesign of service and operations may potentially improve sustainability in economic, environmental, and social dimensions. In this regard, this conceptual framework helps to better understand the adoption of Industry 4.0 technologies in various smart reverse logistics operations. Based on the analysis of the existing literature and case studies, Table 2 presents a technological framework for supporting smart and sustainable reverse logistics transformation. The subsequent sub-sections discuss the potential paradigm changes with respect to the five main reverse logistics processes: (1) smart collection; (2) smart sorting and process management; (3) smart remanufacturing and recycling; (4) smart transportation and distribution; (5) smart disposal.

**Table 2:** Technological framework for supporting smart and sustainable reverse logistics transformation

Industry 4.0 technology	Smart reverse logistics transformation					References
	Smart collection	Smart sorting and process management	Smart remanufacturing and recycling	Smart transportation and distribution	Smart disposal	
IoT/CPS	• IoT-embedded smart bins • Smart monitoring	• Balanced inventory and process management through smart sorting	• Digitized the entire product life cycle	• Dynamic optimization and data-driven fleet management and vehicle routing • Improved traceability	• Intelligent remote-control operations	Chowdhury et al. (2018); Fatimah et al. (2020); Garrido-Hidalgo et al. (2019); Gebresenbet et al. (2018); Gutierrez et al. (2015); John et al. (2021); Liu et al. (2019); Wang and Wang (2019)
Big data	• Smart prediction and monitoring • Real-time routing of collection vehicles		• Predictive planning and real-time decision-makings			Filip and Duta (2015); Ramos et al. (2018); Zhang et al. (2018)
Cloud technology	• Cloud-based autonomous waste collection system			• Effective collaboration, better resource sharing, and demand matching through cloud-based digitalization	• Cloud-based leachate monitoring and management	Cotet et al. (2020); Gebresenbet et al. (2018); Gopikumar et al. (2021)
AR			• Effective functionality restoration and individualized maintenance services			Chang et al. (2017).
AM			• Flexible product redesign and data-driven remanufacturing planning			Kerin and Pham (2019)
Virtual technology	• Dynamic web data dashboard		• Better working procedures through real-time instructions and task visualizations • Predictive planning and real-time and effective decision-makings	• Effective collaboration, better resource sharing, and demand matching through system integration		Gebresenbet et al. (2018);John et al. (2021); Okorie et al. (2020); Thürer et al. (2019)
Autonomous robots	• Smart robots for autonomous waste collection	• AI-enabled intelligent robot-based autonomous sorting system	• Better working procedures and effective functionality restoration			Gundupalli Paulraj et al. (2016); Kumar et al. (2021); Sarc et al. (2019); Wang et al. (2020); Zhang et al. (2019)
AI	• Digital and individualized collection services	• Smart sorting multi-criteria analysis		• Self-driving trucks and automated driving support systems	• Garbage disposal EVs	Kumar et al. (2021); Sung et al. (2020); Wilson et al. (2021)
UAV			• Assist in monitoring the remanufacturing process	• Self-driving trucks		Klumpp (2018)

### Smart collection

Even though the routes can be regularly optimized in a traditional collection system of EOL products, the inherent uncertainty may lead to a resource allocation dilemma, which requires a balance between operating costs and service levels. For instance, the collection of EOL products and other types of waste on fixed schedule and routes usually leads to inefficient use of resources, high fuel consumption (Lu et al. 2020), and low service level. To make it worse, the low service level of biodegrade waste may result in an accumulation of bacteria from bad odors and the spread of diseases (John et al. 2021). To tackle this problem, smart bins embedded with IoT sensors are increasingly used to monitor and provide real-time information about their fill levels and locations (Gutierrez et al. 2015), based on which the collection routes can be dynamically optimized and digitally updated. An IoT-driven Kanban system was designed by Thürer et al. (2019) for the collection of EOL products. Another IoT-enabled prediction and monitoring system was proposed by John et al. (2021), which could be installed in the existing collection bins of different sizes. Empowered by an intelligent neural network, it can learn and predict the waste generation patterns and send timely notifications to appropriate personnel via a firebase cloud messaging system with a dynamic web data dashboard.

Combining with GIS and data-driven optimization models, the routing of collection vehicles can be individualized and dynamically optimized with real-time data (Ramos et al. 2018), based on which the collection service can be drastically improved without an increase in resource needs. To guarantee the real-time capability of data transmission, Cotet et al. (2020) developed a cloud-based automated system for innovative waste collection services. The combination of smart sensors, data, and optimization algorithms forms a smart CPS for EOL product collection in reverse logistics (Bányai et al. 2019). With increasing customers’ involvement via digital platforms, the collection service can be provided based on individualized customer demands (Sung et al. 2020). This provides a new business model for improved policy-making and value proposition, e.g., pricing-by-service, and for better interactions among different stakeholders. In addition, the use of smart robots for autonomous waste collection has recently been focused on during the COVID-19 pandemic due to their potential impacts on reducing infection risks of health workers.

### Smart sorting and process management

Due to the complex composition and quality of EOL products in the reverse flows, sorting is traditionally a semi-automated and labor-intensive process, where different recyclables need to be manually picked up and separated by human workers. However, the hazardous substances and the harsh working environment have put significant threats to the health of these workers. The recent developments of AI and vision-based systems have empowered smart robots with the capability of recognizing and automatically separating different types of recyclables (Wang et al. 2020; Zhang et al. 2019), which has shown great potential to become the gamechanger in reverse logistics operations (Wilson et al. 2021). An automated AI-enabled intelligent robot-based sorting system has been investigated for separating hazardous materials from WEEE (Sarc et al. 2019). For some types of EOL products, i.e., aluminum cans and plastic bottles, recent research shows that the separation accuracy by robot-based smart systems can be up to 90% (Gundupalli Paulraj et al. 2016a). To support the separate collection of different types of EOL products at the sources, e.g., home and office, a prototype of small-scale automatic sorting bins is developed by Ismail et al. (2018), which used smart sensors and material classification technologies.

Industry 4.0 technologies can also help to better manage different processes and facilities. The end-to-end integration of radio-frequency identification (RFID), Bluetooth low energy (BLE), smart sensors, smart containers, and hybrid gateway in a networked CPS allows real-time information collected from various reverse logistics processes, i.e., returned product identification, classification, local information, and global information, which can be used for better inventory control and environmental management of the whole process (Garrido-Hidalgo et al. 2019a). Another reverse logistics challenge is caused by the increased generation of infectious waste during the COVID-19 pandemic (Babaee Tirkolaee and Aydın 2021;Yu et al. 2020), and a large proportion is mixed with conventional waste especially in developing countries (Kumar et al. 2021). Thus, an AI-based automated system is established by Kumar et al. (2021), which provides an integrated solution for more accurate sorting of COVID-19-related medical waste streams from other waste types to support data-driven recycling planning.

### Smart remanufacturing and recycling

From cloud-based systems to digital twins (Wang and Wang 2019), Industry 4.0 paves the way for a data-driven smart remanufacturing process. The high uncertainty related to the quality, quantity, time, and locations of return of EOL products, e.g., WEEE and used vehicles, is the most significant hindrance in a traditional remanufacturing process. To tackle this, a product-based digital twin that integrates IoT and cloud technologies enables smart data collection and condition monitoring throughout the whole product life cycle (Wang and Wang 2019). Besides, consumers can also easily provide relevant product-related information via several digital platforms, e.g., smartphone apps and websites. Based on the generic architecture proposed by Wang and Wang (2019), a personalized digital twin can be developed for tracking the relevant data of specific products, which will be used for better identification, classification, and sorting for further processing.

Big data analytics can help to maximize the value recovery of EOL products through better information on specific production times and options in reverse logistics (Filip and Duta 2015). Using the real-time product information and system data as the dynamic inputs to the optimization models can maximize the effectiveness and resource utilization through improved and more flexible production planning for remanufacturing (Zhang et al. 2018). Besides, a data-driven intelligent dismantling may also reduce the damage during product dissembling and improve the quality and predictability of remanufactured products (Alcayaga et al. 2019). In addition, some other technologies can also help to improve the remanufacturing and recycling operations. For example, the quality and effectiveness of the maintenance service and functionality restoration in remanufacturing can be improved through intuitive step-by-step AR guidance to human operators in a product disassembling process (Chang et al. 2017). Besides, the use of UAVs can assist in monitoring the remanufacturing process. Additive manufacturing provides a more flexible and cost-efficient way to restore the functionalities of EOL products and dissembled components (Kerin and Pham 2019). At the system level, computer-based simulation can provide deep and visualized insights into the system behaviors in a smart remanufacturing process. In this regard, hybrid simulation techniques, i.e., system dynamics, discrete event simulation, and agent-based modeling, are used to investigate the impact of smart technologies as well as the economic viability of remanufacturing (Okorie et al. 2020).

### Smart transportation and distribution

The effective sharing of information and resources is one of the most important features of Industry 4.0, and this provides different stakeholders in a reverse logistics system with the opportunities to better utilize their resources. IoT-based smart platforms have been used for dynamic optimization of demand allocation and routing of transport vehicles (Liu et al. 2019). The real-time vehicle data is collected from GIS, IoT sensors, 4G/5G devices, RFID, and GPS devices, which are then processed to match the task data from different companies. Finally, the assignments and routing decisions are optimized to achieve the most efficient use of available vehicles for multiple assignments from different companies. The system can be further optimized with real-time traffic data for dynamic routing to minimize fuel consumption, greenhouse gas (GHG) emissions, and traffic congestion. A web-based information sharing system is developed by Gebresenbet et al. (2018) for the reverse logistics management of agricultural biomass. The real-time information is collected via both smart devices and end-users, through which the demands and the supplies can be better matched to achieve a high level of inter-company resource utilization. The improved traceability can help to reduce product losses and logistics costs, while at the same time, improving market opportunity and product quality (Gebresenbet et al. 2018). In addition, by connecting cameras, smart sensors, and radar equipment to the network of AI-enabled onboard computers, self-driving trucks have shown a great potential to realize autonomous driving (Wilson et al. 2021). In some tasks, smart AI has already overtaken human competence levels, and with the continuous maturity of autonomous vehicle technology, the paradigm of reverse logistics will also be largely changed in the near future (Klumpp 2018).

### Smart disposal

The problem of waste disposal is not only related to dealing with the disposal of waste in the proper place but is also associated with reducing the volume of waste disposal (Karnalim et al. 2020), safety issues, and cleanliness (Fernandes and Wairkar 2020). Even though an increasing amount of EOL products are recycled, incineration plants and landfills are still the final destinations of the non-recyclables in reverse logistics systems, where smart robots can be used to replace human workers in harsh working environments. IoT-enabled smart systems can help to monitor the key performance indicators and remotely control different operations (Chowdhury et al. 2018; Fatimah et al. 2020). The landfill of solid waste generates landfill gas and high-density hazardous liquid, called leachate, both of which have significant environmental impacts and need thus to be properly treated. To better manage the leachate problems, a cloud-based IoT system can play an important role in connecting the relevant field data with respective mathematical models to analyze several key parameters, i.e., turbidity, suspended solids, and dissolved oxygen, for smart disposal (Gopikumar et al. 2021). Besides, the smart bin is a solution for convenient waste disposal without the need to touch the lid, which avoids the spread of disease especially during the pandemic (Fernandes and Wairkar 2020).

## A future research agenda

**Fig. 6:**
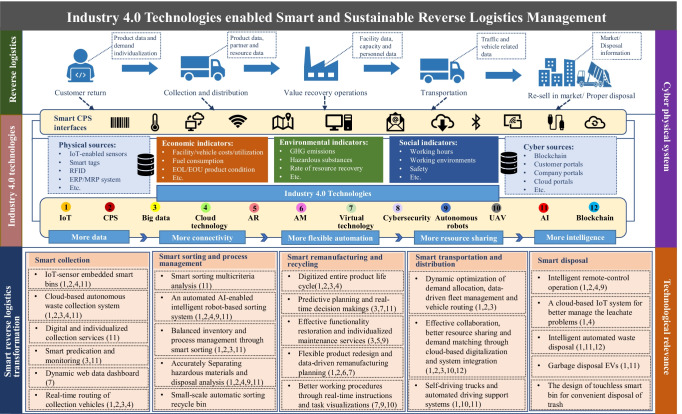
The architecture of the smart reverse logistics system enabled by Industry 4.0

By analyzing the potential impacts of Industry 4.0 technologies on reverse logistics processes, Fig. 6 illustrates the architecture that enables the smart and sustainable transformation in *Reverse Logistics 4.0*. The smart and sustainable reverse logistics transformation concisely presents the union of both physical and digital value chains. On the one hand, a physical value chain illustrates the application and impacts of these disruptive technologies at both inter- and intra-organizational levels. On the other hand, a digital value chain assesses the long-term impacts on value-adding and value recovery patterns from a technological standpoint (Tozanlı and Kongar 2020). The proposed architecture explicitly links reverse logistics activities, Industry 4.0-enabled cyber-physical connection and interaction, and technological enablers for the smart transformation of collection, sorting and process management, remanufacturing and recycling, transportation and distribution, and waste disposal. It is noteworthy that the targeted sustainability goals are centered on the architecture of smart reverse logistics transformation, which further reflects the ultimate goal of *Reverse Logistics 4.0* is not to adopt technology but to improve sustainability through service and operation transformation by using technology, as shown in Fig. 5. In this regard, Industry 4.0 technologies can provide more data, more connectivity, more intelligence, more flexible automation, and better resource sharing, through which the sustainable goals can be better archived through the improvement of various reverse logistics activities and processes. In addition, based on the analysis of reported research and cases in the literature, Fig. 6 illustrates a mapping between the smart reverse logistics transformation and the proper Industry 4.0 enablers.

Even though recent research efforts have been increasingly given to Industry 4.0-enabled smart and sustainable reverse logistics (Dev et al. 2020a;Dev et al. 2020b), there are still several gaps, e.g., a lack of comprehension and understanding of Industry 4.0 (Pourmehdi et al. 2021), unclear benefits, and lack of quantitative approach for performance evaluation (Sun et al. 2021), that need to be filled. Thus, we identified four directions for guiding future research:

- *Smart and innovative reverse logistics services*: Service innovation is the most important driver for the paradigm shift in *Reverse Logistics 4.0* to better meet the sustainability goals. However, the current research puts a predominant focus on the smart operations of isolated reverse logistics activities but not on the service innovation, which consequently hinders the real-world adoption of Industry 4.0 technologies due to the unclear benefits on customer value (Pourmehdi et al. 2022). Furthermore, the role of customers in the smart reverse logistics transformation has not been well investigated (Shokouhyar et al. 2021). Even though digital platforms are widely used today for better information sharing between customers and collection companies, for example, on the collection schedules of different types of EOL products and waste, they are mainly a one-way information flow and customers cannot require individualized collection service based on their actual needs. Thus, research needs to be carried out to better understand how Industry 4.0 technologies can be used to effectively and efficiently meet individualized demands in reverse logistics, which opens several research possibilities, e.g., demand/data-driven collection service systems, new business models, and pricing strategies for the value proposition through demand individualization and service diversification. In addition, a cloud-based system can provide a platform for end-users to register the relevant information of the returned EOL products (Wang and Wang 2019), but the supporting policies and mechanisms have not been well in place to promote the customers’ active participation in reverse logistics. In this regard, future research is invited to focus on service innovation and smartness, as well as the customers’ role and active involvement in *Reverse Logistics 4.0*, through which the “service-based individualization” can be better interpreted to show clearer customer value and benefits to the companies. It will eventually help to promote the smart and sustainable transformation of *Reverse Logistics 4.0*.
- *Quantitative models for smart and sustainable reverse logistics management*: The Industry 4.0-enabled smart reverse logistics services will lead to a transformation of traditional reverse logistics operations by increasing connectivity, smartness, and autonomous operations. Thus, there is a need for new quantitative models or new ways of using and integrating existing models to deal with new challenges (Olsen and Tomlin 2020), e.g., predictive operational planning with AI and real-time data integration, and to better support strategic, tactical, and operational decisions for smart and sustainable reverse logistics management. For example, reverse logistics network design is one of the most important strategic designs, which may yield long-term impacts on sustainable performance. The smart transformation in *Reverse Logistics 4.0* may dramatically change the operations and the key parameters within the planning horizon, which makes the initial network design becoming much more complex. In addition, implementing Industry 4.0 technologies to reduce internal operating costs through digital end-to-end integration is complex and requires a large initial investment (Bai et al. 2020), so a holistic analysis is needed to understand the long-term impacts of this smart transformation. In this regard, new quantitative models and methods are needed for better decision support and comprehensive scenario analyses of the potential impact of smart reverse logistics transformation in the strategic network design, which can provide holistic insights to the decision-makers.
- *Digital reverse logistics twin*: As shown in Fig. 6, the combination of the physical world and the cyber world in a smart digital twin is a promising research direction in *Reverse Logistics 4.0*, where, for example, AI-enabled data prediction and real-time data collected from both cyber and physical sources can be collaboratively used with mathematical models and computer-based simulation to better predict the key parameters or the parameter distributions for the quantitative decision support models (Ivanov and Dolgui 2021), which will help to minimize the impact of uncertainty in the reverse flows and to yield robust decisions, e.g., scheduling and vehicle routing (Zhang et al. 2018). The high-quality visualization of the reverse logistics system can help decision-makers to better analyze different operations. Furthermore, developing bi-directional control and interactions of the smart digital twin provides opportunities for autonomous reverse logistics operations. A smart digital reverse logistics twin requires an in-depth methodological integration and a high-level system integration, where various smart robots and devices, software, data, analytical models, visualization tools, etc. need to be effectively and seamlessly connected and interacted (Sun et al. 2022). Furthermore, due to the complex flows and the involvement of several stakeholders, the system boundary of the smart transformation needs to be clearly defined, which helps to better interact with different reverse logistics players. In addition, multiple sustainability indicators need to be measured with both cyber and physical sources and be accounted for in the quantitative models for decision support. For example, the real-time routing may be dynamically optimized considering several objectives to balance economic costs, truck utilization, GHG emissions, and driver’s working time.
- *Human-centricity and Reverse Logistics 5.0*: Even though the opportunities for improving sustainability and circular economy have been discussed, Industry 4.0 is primarily a technology-driven paradigm shift. The recently proposed concept of Industry 5.0 has led to a changing focus from technology to human-centricity, resilience, and sustainability in the transition of many sectors (Frederico 2021;Jafari et al. 2022), which may result in new research directions for smart reverse logistics transformation. For instance, Industry 4.0 focuses on the development of autonomous solutions to replace human workers. However, on the other hand, Industry 5.0 emphasizes the harmony between humans and technology, where technologies are used not to replace humans but to better help human workers and create new job opportunities (Jafari et al. 2022). In this regard, future research is invited to further investigate the human-centric, resilient, and sustainable transformation of *Reverse Logistics 5.0*. Some specific topics are, for example, the updated sustainability goals in *Reverse Logistics 5.0*, especially from the social and environmental perspectives, human-machine collaboration in reverse logistics, the use of AR and collaborative robots (Cobot) for various operations (Ramírez et al. 2020), and so forth.

Table 3 presents several promising topics to better guide future research in each direction.

**Table 3:** Future research agenda

Research directions	Specific topics
Smart and innovative reverse logistics services	• Demand/data-driven waste collection service • New business models for value proposition through individualized and diversified services • Pricing strategies for individualized collection service • Customers’ role in smart and sustainable reverse logistics transformation • Supporting mechanisms for promoting end-users’ participation in reverse logistics
Quantitative models for smart and sustainable reverse logistics management	• Quantitative methods for evaluating the impacts of Industry 4.0 technologies, e.g., IoT, AI, additive manufacturing, smart robots, etc., on smart reverse logistics operations • Smart and sustainable reverse logistics network design • Data-driven proactive reverse logistics operational planning with AI and optimization (e.g., remanufacturing and recycling) • Data-driven dynamic and real-time vehicle routing for collection and transportation of EOL products (traffic data, fill level, etc.)
Digital reverse logistics twin	• Product-based digital twin with IoT and cloud technologies for data collection in the EOL stage • Methodological integration (predictive analytics, prescriptive analytics, and descriptive analytics) • Cyber-physical system integration (IoT sensors, smart devices, data, analytical models, and algorithms) • Real-time decision support and optimization under multiple sustainability goals
Human-centricity and *Reverse Logistics 5.0*	• Definition and conceptualization of the human-centric smart transformation of *Reverse Logistics 5.0* and the updated sustainability goals • The role of humans in the paradigm transition of reverse logistics • The development and use of collaborative technologies in smart reverse logistics systems • The impacts of adopting collaborative technologies in smart reverse logistics service and operations

## Conclusion

Today, Industry 4.0 provides new opportunities and solutions to combine physical elements and data, autonomous technologies, internet- and cloud-based connectivity, data-driven analytics, and model-based analytics in highly digitalized and smart reverse logistics systems. However, there is still a lack of a systematic conceptualization to guide the paradigm transition of reverse logistics in Industry 4.0. Therefore, based on the reported research and case studies from the literature, this paper aims at contributing to the definition and conceptual development of *Reverse Logistics 4.0* and providing a general framework of the smart reverse logistics transformation to better achieve the sustainability goals in the triple-bottom-line by answering the three research questions:

- To answer *RQ1*, the theoretical and practical evolvement of the concept of reverse logistics is discussed in comparison with the four Industrial Revolutions in history. *Reverse Logistics 4.0* is then defined based on the paradigm shift brought by Industry 4.0.
- To answer *RQ2*, the general conceptual framework for the smart reverse logistics transformation is proposed considering technological enablers, smart service transformation, smart operation transformation, and sustainability goals. Moreover, the implications of adopting Industry 4.0 technologies in smart collection, smart sorting and process management, smart remanufacturing and recycling, smart transportation and distribution, and smart disposal are thoroughly analyzed.
- To answer *RQ3*, a research agenda with four research directions is given to show the roadmap towards *Reverse Logistics 4.0* through smart and sustainable transformation, and several specific topics are also suggested for each research direction.

### *Research implications*

This paper provides a systematic definition, conceptualization, and research agenda of *Reverse Logistics 4.0* to thoroughly link Industry 4.0, reverse logistics, and sustainability goals in the smart paradigm transition. Furthermore, research opportunities are clearly identified to guide future theoretical and methodological developments related to smart reverse logistics service innovation, quantitative models for smart and sustainable reverse logistics management, digital reverse logistics twin, and human-centricity and *Reverse Logistics 5.0*.

### *Managerial implications*

This paper provides a conceptual framework that can help decision-makers and practitioners to understand how these sustainability goals can be better met through the technology-driven smart service and operation transformations of a reverse logistics system. Furthermore, it also presents a mapping between the technological enablers in Industry 4.0 and the smart transformation of different reverse logistics processes, and this provides a guide for the technology adoption of reverse logistics companies.

### *Future works*

Even though research efforts have been spent to develop smart reverse logistics planning, especially with the application of real-time data in several operations, there is still a need for a better understanding of service innovation, customer participation, and the role of humans, as well as other key influencing factors in *Reverse Logistics 4.0*. The impacts of smart reverse logistics transformation need to be holistically and comprehensively taken into account in the initial planning stage, e.g., network design. Besides, increased methodological integration and system integration are needed to realize the concept of a highly integrated and intelligent digital reverse logistics twin. Future research is thus invited to tackle these challenges.

## Data Availability

Not applicable.

## References

[CR1] Afra AP, Behnamian J (2021). Lagrangian heuristic algorithm for green multi-product production routing problem with reverse logistics and remanufacturing. J Manuf Syst.

[CR2] Agrawal S, Singh RK, Murtaza Q (2015). A literature review and perspectives in reverse logistics. Resour Conserv Recycl.

[CR3] Akinlar S (2014) Logistics 4.0 and challenges for the supply chain planning and IT. Fraunhofer IML. https://www.iis.fraunhofer.de/content/dam/iis/tr/Session%203_5_Logistics_Fraunhofer%20IML_Akinlar.pdf (accessed on 05.05.2022)

[CR4] Alcayaga A, Wiener M, Hansen EG (2019). Towards a framework of smart-circular systems: an integrative literature review. J Clean Prod.

[CR5] Alshamsi A, Diabat A (2017). A genetic algorithm for reverse logistics network design: a case study from the GCC. J Clean Prod.

[CR6] Atasu A, Guide VDR, Van Wassenhove LN (2010). So what if remanufacturing cannibalizes my new product sales?. Calif Manag Rev.

[CR7] Babaee Tirkolaee E, Aydın NS (2021). A sustainable medical waste collection and transportation model for pandemics. Waste Manag Res.

[CR8] Bag S, Telukdarie A, Pretorius J, Gupta S (2018). Industry 4.0 and supply chain sustainability: framework and future research directions. Benchmarking Intl J.

[CR9] Bag S, Gupta S, Kumar S (2021). Industry 4.0 adoption and 10R advance manufacturing capabilities for sustainable development. Int J Prod Econ.

[CR10] Bai C, Dallasega P, Orzes G, Sarkis J (2020). Industry 4.0 technologies assessment: a sustainability perspective. Int J Prod Econ.

[CR11] Bányai T, Tamás P, Illés B, Stankevičiūtė Ž, Bányai Á (2019). Optimization of municipal waste collection routing: impact of industry 4.0 technologies on environmental awareness and sustainability. Int J Environ Res Public Health.

[CR12] Barreto L, Amaral A, Pereira T (2017). Industry 4.0 implications in logistics: an overview. Procedia Manuf.

[CR13] Beiler BC, de Arruda Ignácio PS, Júnior ACP, Anholon R, Rampasso IS (2020). Reverse logistics system analysis of a Brazilian beverage company: an exploratory study. J Clean Prod.

[CR14] Bradu P, Biswas A, Nair C, Sreevalsakumar S, Patil M, Kannampuzha S, Mukherjee AG, Wanjari UR, Renu K, Vellingiri B (2022) Recent advances in green technology and Industrial Revolution 4.0 for a sustainable future. Environ Sci Pollut Res:1–32. 10.1007/s11356-022-20024-4 10.1007/s11356-022-20024-4 PMC8994424 35397034

[CR15] Calvo-Porral C, Lévy-Mangin J-P (2020). The circular economy business model: examining consumers’ acceptance of recycled goods. Adm Sci.

[CR16] Chang M, Ong S, Nee A (2017). AR-guided product disassembly for maintenance and remanufacturing. Procedia CIRP.

[CR17] Chowdhury P, Sen R, Ray D, Roy P, Sarkar S (2018) Garbage monitoring and disposal system for smart city using IoT, 2018 Second International Conference on Green Computing and Internet of Things (ICGCIoT). IEEE, 455-460. 10.1109/ICGCIoT.2018.8753060

[CR18] Cotet CE, Deac GC, Deac CN, Popa CL (2020). An innovative industry 4.0 cloud data transfer method for an automated waste collection system. Sustainability.

[CR19] de Paula IC, de Campos EAR, Pagani RN, Guarnieri P, Kaviani MA (2019). Are collaboration and trust sources for innovation in the reverse logistics? Insights from a systematic literature review. Supply Chain Manag.

[CR20] Dev NK, Shankar R, Qaiser FH (2020). Industry 4.0 and circular economy: operational excellence for sustainable reverse supply chain performance. Resour Conserv Recycl.

[CR21] Dev NK, Shankar R, Swami S (2020). Diffusion of green products in industry 4.0: reverse logistics issues during design of inventory and production planning system. Int J Prod Econ.

[CR22] Diabat A, Abdallah T, Al-Refaie A, Svetinovic D, Govindan K (2013). Strategic closed-loop facility location problem with carbon market trading. IEEE Trans Eng Manag.

[CR23] Dowlatshahi S (2000). Developing a theory of reverse logistics. Interfaces.

[CR24] Duberg JV, Johansson G, Sundin E, Kurilova-Palisaitiene J (2020). Prerequisite factors for original equipment manufacturer remanufacturing. J Clean Prod.

[CR25] Facchini F, Oleśków-Szłapka J, Ranieri L, Urbinati A (2020). A maturity model for logistics 4.0: an empirical analysis and a roadmap for future research. Sustainability.

[CR26] Fallahpour A, Wong KY, Rajoo S, Fathollahi-Fard AM, Antucheviciene J, Nayeri S (2021) An integrated approach for a sustainable supplier selection based on Industry 4.0 concept. Environ Sci Pollut Res 1-19. 10.1007/s11356-021-17445-y 10.1007/s11356-021-17445-y 34792774

[CR27] Fatimah YA, Govindan K, Murniningsih R, Setiawan A (2020). Industry 4.0 based sustainable circular economy approach for smart waste management system to achieve sustainable development goals: a case study of Indonesia. J Clean Prod.

[CR28] Fattahi M, Govindan K (2017). Integrated forward/reverse logistics network design under uncertainty with pricing for collection of used products. Ann Oper Res.

[CR29] Fernandes Y, Wairkar S (2020). Safe waste disposal using smart dustbin. Int Res J Eng Technol.

[CR30] Filip FG, Duta L (2015). Decision support systems in reverse supply chain management. Procedia Econ Financ.

[CR31] Fleischmann M, Bloemhof-Ruwaard JM, Dekker R, Van der Laan E, Van Nunen JA, Van Wassenhove LN (1997). Quantitative models for reverse logistics: a review. Eur J Oper Res.

[CR32] Ford S, Despeisse M (2016). Additive manufacturing and sustainability: an exploratory study of the advantages and challenges. J Clean Prod.

[CR33] Frank AG, Dalenogare LS, Ayala NF (2019). Industry 4.0 technologies: implementation patterns in manufacturing companies. Int J Prod Econ.

[CR34] Frederico GF (2021). From supply chain 4.0 to supply chain 5.0: findings from a systematic literature review and research directions. Logistics.

[CR35] Garrido-Hidalgo C, Olivares T, Ramirez FJ, Roda-Sanchez L (2019). An end-to-end internet of things solution for reverse supply chain management in industry 4.0. Comput Ind.

[CR36] Gebresenbet G, Bosona T, Olsson S-O, Garcia D (2018). Smart system for the optimization of logistics performance of the pruning biomass value chain. Appl Sci.

[CR37] Gonçalves ATT, Fagundes LD, Miranda RC, Lima RS (2019). Discrete event simulation as a decision-making tool for end-of-life tire reverse logistics in a Brazilian city consortium. Environ Sci Pollut Res.

[CR38] Gopikumar S, Raja S, Robinson YH, Shanmuganathan V, Chang H, Rho S (2021). A method of landfill leachate management using internet of things for sustainable smart city development. Sustain Cities Soc.

[CR39] Govindan K, Soleimani H, Kannan D (2015). Reverse logistics and closed-loop supply chain: a comprehensive review to explore the future. Eur J Oper Res.

[CR40] Govindan K, Paam P, Abtahi A-R (2016). A fuzzy multi-objective optimization model for sustainable reverse logistics network design. Ecol Indic.

[CR41] Govindan K, Bouzon M (2018). From a literature review to a multi-perspective framework for reverse logistics barriers and drivers. J Clean Prod.

[CR42] Gu W, Wei L, Zhang W, Yan X (2019). Evolutionary game analysis of cooperation between natural resource-and energy-intensive companies in reverse logistics operations. Int J Prod Econ.

[CR43] Gundupalli Paulraj S, Hait S, Thakur A (2016) Automated municipal solid waste sorting for recycling using a mobile manipulator, International design engineering technical conferences and computers and information in engineering conference. American Society of Mechanical Engineers, Charlotte, North Carolina, USA. V05AT07A045. 10.1115/DETC2016-59842

[CR44] Gutierrez JM, Jensen M, Henius M, Riaz T (2015). Smart waste collection system based on location intelligence. Procedia Comput Sci.

[CR45] Ivanov D, Dolgui A (2021). A digital supply chain twin for managing the disruption risks and resilience in the era of Industry 4.0. Prod Plan Control.

[CR46] Ismail INb, Jayakumar P, Eqwan M, Zuhdi AWM, Mohamad D, Isa MR, Zahari NM, Zawawi MH, Mohamed H, Ramli MZ (2018) Design and development of smart sorting recycle bin prototype, AIP Conference Proceedings. AIP Publishing LLC, 020202. 10.1063/1.5066843

[CR47] Jafari N, Azarian M, Yu H (2022). Moving from Industry 4.0 to Industry 5.0: what are the implications for smart logistics?. Logistics.

[CR48] John J, Varkey MS, Podder RS, Sensarma N, Selvi M, Santhosh Kumar S, Kannan A (2021). Smart prediction and monitoring of waste disposal system using IoT and cloud for IoT based smart cities. Wirel Pers Commun.

[CR49] Julianelli V, Caiado RGG, Scavarda LF, Cruz SPMF (2020). Interplay between reverse logistics and circular economy: critical success factors-based taxonomy and framework. Resour Conserv Recycl.

[CR50] Karnalim O, Wongso O, Budiman VE, Jonathan FC, Manuel BA, Marlina M (2020). A persuasive technology for managing waste disposal through smart trash bin and waste disposal tracker. Int J Inf Commun Technol.

[CR51] Kerin M, Pham DT (2019). A review of emerging industry 4.0 technologies in remanufacturing. J Clean Prod.

[CR52] Klumpp M (2018). Automation and artificial intelligence in business logistics systems: human reactions and collaboration requirements. Int J Log Res Appl.

[CR53] Kumar NM, Mohammed MA, Abdulkareem KH, Damasevicius R, Mostafa SA, Maashi MS, Chopra SS (2021). Artificial intelligence-based solution for sorting COVID related medical waste streams and supporting data-driven decisions for smart circular economy practice. Process Saf Environ Prot.

[CR54] Lambert S, Riopel D, Abdul-Kader W (2011). A reverse logistics decisions conceptual framework. Comput Ind Eng.

[CR55] Lasi H, Fettke P, Kemper H-G, Feld T, Hoffmann M (2014). Industry 4.0. Bus Inf Syst Eng.

[CR56] Lee J, Bagheri B, Kao H-A (2015). A cyber-physical systems architecture for industry 4.0-based manufacturing systems. Manuf Lett.

[CR57] Liu B, De Giovanni P (2019) Green process innovation through Industry 4.0 technologies and supply chain coordination. Ann. Oper Res 1-36. 10.1007/s10479-019-03498-3

[CR58] Liu S, Zhang Y, Liu Y, Wang L, Wang XV (2019). An ‘Internet of Things’ enabled dynamic optimization method for smart vehicles and logistics tasks. J Clean Prod.

[CR59] Lu X, Pu X, Han X (2020). Sustainable smart waste classification and collection system: a bi-objective modeling and optimization approach. J Clean Prod.

[CR60] Okorie O, Charnley F, Ehiagwina A, Tiwari D, Salonitis K (2020) Towards a simulation-based understanding of smart remanufacturing operations: a comparative analysis. J Remanufac 1-24. 10.1007/s13243-020-00086-8

[CR61] Oleśków-Szłapka J, Stachowiak A (2018) The framework of logistics 4.0 maturity model, International conference on intelligent systems in production engineering and maintenance. Springer, 771-781. 10.1007/978-3-319-97490-3_73

[CR62] Olsen TL, Tomlin B (2020). Industry 4.0: opportunities and challenges for operations management. Manuf Serv Oper Manag.

[CR63] Pandian GRS, Abdul-Kader W (2017). Performance evaluation of reverse logistics enterprise–an agent-based simulation approach. Int J Sustain Eng.

[CR64] Phuyal S, Bista D, Bista R (2020). Challenges, opportunities and future directions of smart manufacturing: a state of art review. Sustain Futures.

[CR65] Plaza-Úbeda JA, Abad-Segura E, Burgos-Jiménez J, Boteva-Asenova A, Belmonte-Ureña LJ (2021). Trends and new challenges in the green supply chain: the reverse logistics. Sustainability.

[CR66] Pourmehdi M, Paydar MM, Ghadimi P, Azadnia AH (2021). Analysis and evaluation of challenges in the integration of Industry 4.0 and sustainable steel reverse logistics network. Comput Ind Eng.

[CR67] Ramírez FJ, Castellani M, Xu W (2020) Autonomous remanufacturing. Int J Adv Manuf Technol 1-2. 10.1007/s00170-020-05559-5

[CR68] Ramos TRP, Gomes MI, Barbosa-Póvoa AP (2014). Planning a sustainable reverse logistics system: balancing costs with environmental and social concerns. Omega.

[CR69] Ramos TRP, de Morais CS, Barbosa-Póvoa AP (2018). The smart waste collection routing problem: alternative operational management approaches. Expert Syst Appl.

[CR70] Ravi V, Shankar R (2015). Survey of reverse logistics practices in manufacturing industries: an Indian context. Benchmarking Intl J.

[CR71] Rogers DS, Tibben-Lembke R (2001). An examination of reverse logistics practices. J Bus Logist.

[CR72] Salema MIG, Barbosa-Povoa AP, Novais AQ (2007). An optimization model for the design of a capacitated multi-product reverse logistics network with uncertainty. Eur J Oper Res.

[CR73] Salkin C, Oner M, Ustundag A, Cevikcan E (2018) A conceptual framework for Industry 4.0, Industry 4.0: managing the digital transformation. Springer, 3-23. 10.1007/978-3-319-57870-5_1

[CR74] Sarc R, Curtis A, Kandlbauer L, Khodier K, Lorber K, Pomberger R (2019). Digitalisation and intelligent robotics in value chain of circular economy oriented waste management–a review. Waste Manag.

[CR75] Sarkis J, Kouhizadeh M, Zhu QS (2020). Digitalization and the greening of supply chains. Ind Manag Data Syst.

[CR76] Senthil S, Murugananthan K, Ramesh A (2018). Analysis and prioritisation of risks in a reverse logistics network using hybrid multi-criteria decision making methods. J Clean Prod.

[CR77] Shokouhyar S, Dehkhodaei A, Amiri B (2021) Toward customer-centric mobile phone reverse logistics: using the DEMATEL approach and social media data. Kybernetes. https://www.emerald.com/insight/content/doi/10.1108/K-11-2020-0831/full/html

[CR78] Smith-Gillespie A, Muñoz A, Morwood D, Aries T (2018) ROLLS-ROYCE: a circular economy business model case. http://hdl.handle.net/10347/20428 (accessed on 05.05.2022)

[CR79] Soleimani H, Mohammadi M, Fadaki M, Mirzapour Al-e-hashem SMJ (2021) Carbon-efficient closed-loop supply chain network: an integrated modeling approach under uncertainty. Environ Sci Pollut Res 1-16. 10.1007/s11356-021-15100-0 10.1007/s11356-021-15100-0 34480699

[CR80] Sun X, Yu H, Solvang WD, Wang Y, Wang K (2021). The application of Industry 4.0 technologies in sustainable logistics: a systematic literature review (2012–2020) to explore future research opportunities. Environ Sci Pollut Res.

[CR81] Sun X, Yu H, Solvang WD (2022) System integration for smart reverse logistics management, 2022 IEEE/SICE International Symposium on System Integration (SII). IEEE, 821-826. 10.1109/SII52469.2022.9708743

[CR82] Sung S-I, Kim Y-S, Kim H-S (2020). Study on reverse logistics focused on developing the collection signal algorithm based on the sensor data and the concept of Industry 4.0. Appl Sci.

[CR83] Thürer M, Pan Y, Qu T, Luo H, Li C, Huang GQ (2019). Internet of things (IoT) driven kanban system for reverse logistics: solid waste collection. J Intell Manuf.

[CR84] Tozanlı Ö, Kongar E (2020) Integration of industry 4.0 principles into reverse logistics operations for improved value creation: a case study of a mattress recycling company. Enterprise & Business Management: A Handbook for Educators, Consultants, and Practitioners; Erkollar, A., Ed, pp 1-26

[CR85] Trochu J, Chaabane A, Ouhimmou M (2018). Reverse logistics network redesign under uncertainty for wood waste in the CRD industry. Resour Conserv Recycl.

[CR86] Velis CA, Wilson DC, Cheeseman CR (2009). 19th century London dust-yards: a case study in closed-loop resource efficiency. Waste Manag.

[CR87] Wang K (2016) Logistics 4.0 solution-new challenges and opportunities, 6th International Workshop of Advanced Manufacturing and Automation. Atlantis Press, 68-74. 10.2991/iwama-16.2016.13

[CR88] Wang L, Wang XV, Gao L, Váncza J (2014). A cloud-based approach for WEEE remanufacturing. CIRP Ann.

[CR89] Wang XV, Wang L (2019). Digital twin-based WEEE recycling, recovery and remanufacturing in the background of Industry 4.0. Int J Prod Res.

[CR90] Wang Z, Li H, Yang X (2020). Vision-based robotic system for on-site construction and demolition waste sorting and recycling. J Build Eng.

[CR91] Waqas M, Dong Q-l, Ahmad N, Zhu Y, Nadeem M (2018). Critical barriers to implementation of reverse logistics in the manufacturing industry: a case study of a developing country. Sustainability.

[CR92] Wilson M, Paschen J, Pitt L (2021). The circular economy meets artificial intelligence (AI): understanding the opportunities of AI for reverse logistics. Manag Environ Qual.

[CR93] Winkelhaus S, Grosse EH (2020). Logistics 4.0: a systematic review towards a new logistics system. Int J Prod Res.

[CR94] Wrobel-Lachowska M, Wisniewski Z, Polak-Sopinska A (2017): The role of the lifelong learning in logistics 4.0, international conference on applied human factors and ergonomics. Springer, Cham, 402-409. 10.1007/978-3-319-60018-5_39

[CR95] Yu H, Solvang W (2016). A stochastic programming approach with improved multi-criteria scenario-based solution method for sustainable reverse logistics design of waste electrical and electronic equipment (WEEE). Sustainability.

[CR96] Yu H, Solvang WD (2017) Enhancing the competitiveness of manufacturers through Small-scale Intelligent Manufacturing System (SIMS): a supply chain perspective, 2017 6th International Conference on Industrial Technology and Management (ICITM). IEEE, 101-107. 10.1109/ICITM.2017.7917904

[CR97] Yu H, Solvang WD (2018). Incorporating flexible capacity in the planning of a multi-product multi-echelon sustainable reverse logistics network under uncertainty. J Clean Prod.

[CR98] Yu H, Sun X, Solvang WD, Zhao X (2020). Reverse logistics network design for effective management of medical waste in epidemic outbreaks: insights from the coronavirus disease 2019 (COVID-19) outbreak in Wuhan (China). Int J Environ Res Public Health.

[CR99] Zhang Y, Liu S, Liu Y, Yang H, Li M, Huisingh D, Wang L (2018). The ‘Internet of Things’ enabled real-time scheduling for remanufacturing of automobile engines. J Clean Prod.

[CR100] Zhang, Z., Wang, H., Song, H., Zhang, S., Zhang, J. (2019) Industrial robot sorting system for municipal solid waste. In: Yu, H., Liu, J., Liu, L., Ju, Z., Liu, Y., Zhou, D. (eds) Intelligent Robotics and Applications. ICIRA 2019. Lecture Notes in Computer Science, 11741. Springer, Cham. 10.1007/978-3-030-27532-7_31

